# Arthroscopic Cartilage Repair of the Patella With Minced Cartilage and Collagen Membrane Scaffold With Bone Marrow Aspirate Concentrate

**DOI:** 10.1016/j.eats.2024.103308

**Published:** 2024-11-13

**Authors:** Edmund Jia Xi Zhang, Zachariah Gene Wing Ow, Edward Vincentius Lie, Ian Dominic Dhanaraj, Keng Lin Wong

**Affiliations:** a Yong Loo Lin School of Medicine, National University of Singapore, Singapore; b Department of Orthopaedic Surgery, Sengkang General Hospital, Singapore; c Department of Orthopaedic Surgery, Woodlands Health, Singapore; d Musculoskeletal Sciences Academic Clinical Programme, Duke-NUS Graduate Medical School, Singapore

## Abstract

The single-staged repair technique of patellofemoral cartilage lesions has previously been described, with the utility of a bilayer scaffold and minced cartilage via an open mini-arthrotomy, showing great clinical efficacy. We describe a procedure similarly aimed at addressing patellofemoral lesions but with an all-arthroscopic approach, using a porcine-derived collagen I/III bilayer scaffold augmented with bone marrow aspirate concentrate and minced cartilage.

Single-staged cartilage-repair procedures have been gaining popularity compared with staged procedures such as autologous chondrocyte implantation because of the benefits of avoiding multiple surgeries while displaying comparable outcomes.^1^ A leading technique of current single-staged techniques is autologous matrix−induced chondrogenesis (AMIC). The AMIC technique describes cartilage repair using an acellular porcine-derived collagen I/III bilayered scaffold, the Chondro-Gide (Geistlich Pharma AG, Wolhusen, Switzerland), with a subsequent evolution termed AMIC+, wherein the cartilage repair expands to involve the use of the scaffold with bone marrow aspirate concentrate (BMAC) as a form of biologic augmentation. We have previously described an all-arthroscopic technique of cartilage repair using this AMIC+ procedure, aimed at addressing patellofemoral articular lesions.^2^ The use of a scaffold with minced cartilage has been reported to effectively address cartilage lesions of the knee with significant improvements in pain and function at 5 years.^3^ In this article, we present the all-arthroscopic cartilage repair of the patella using the AMIC+ procedure with minced cartilage augmentation (Video 1).

## Surgical Technique

### Patient Evaluation, Imaging, and Indications

Concomitant conditions like ligamentous insufficiencies/ruptures or uncorrected malalignments of the joint should be ruled out.^4^ Preoperative imaging such as radiographs and magnetic resonance imaging should be obtained to evaluate alignment and characterize the cartilage lesion. Cartilage defects that would be a candidate for repair should be critically sized, contained defects surrounded by adequate healthy cartilage.^2^

### Patient Positioning and Anesthesia

The patient is positioned supine as for standard knee arthroscopy with the foot attachment removed for increased access to the knee joint. A tourniquet is applied high on the thigh.

### Diagnostic Arthroscopy

Landmarks of the knee joint are marked. These landmarks include the inferior pole and borders of the patellar tendon, medial and lateral joint lines, and tibial tuberosity. Standard anteromedial and anterolateral portals are then made, with an additional accessory working portal being created as well via a medial approach to the suprapatellar pouch. Normal saline is instilled into the joint space and routine diagnostic arthroscopy is performed with a 30° offset arthroscope being used to visualize all knee compartments and locate the osteochondral injury on the patella. The lesion is probed and staged, and loose osteochondral fragments floating within the joint space are removed.

### Preparation of the Scaffold and BMAC

**Fig 1:**
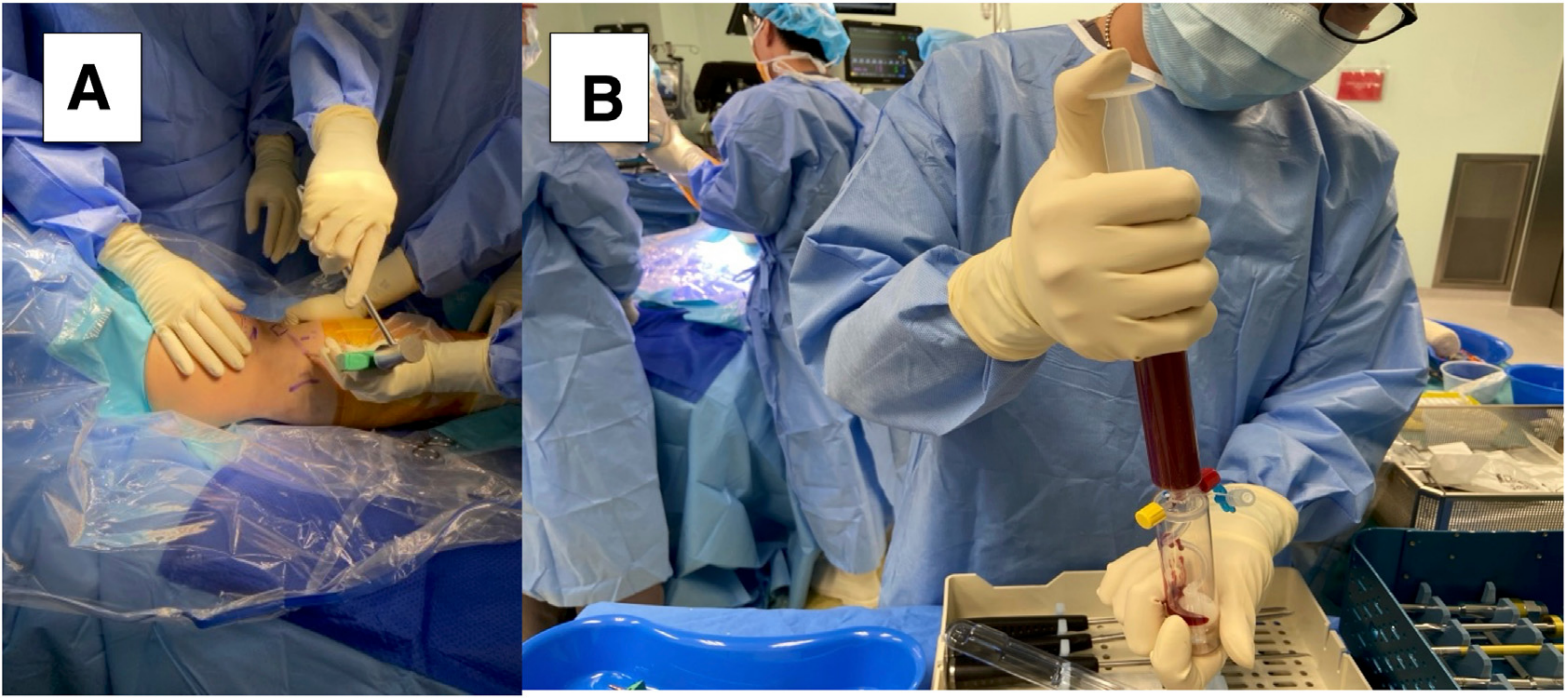
(A) Harvesting of 30 mL of bone marrow aspirate is performed using a bone marrow–aspiration trocar (BioCUE BMA) at the ipsilateral right proximal tibia. (B) The bone marrow aspirate is delivered into the collection system to be centrifuged.

We use the BioCUE BMA Concentration System (Zimmer Biomet, Warsaw, IN). At the ipsilateral proximal tibia, a bone marrow aspiration trocar is inserted to aspirate 30 mL of bone marrow (Fig 1). The aspiration is centrifuged at 3200 rpm for 15 minutes with the GPS III system (Zimmer Biomet) to produce approximately 3 mL of BMAC.

**Fig 2:**
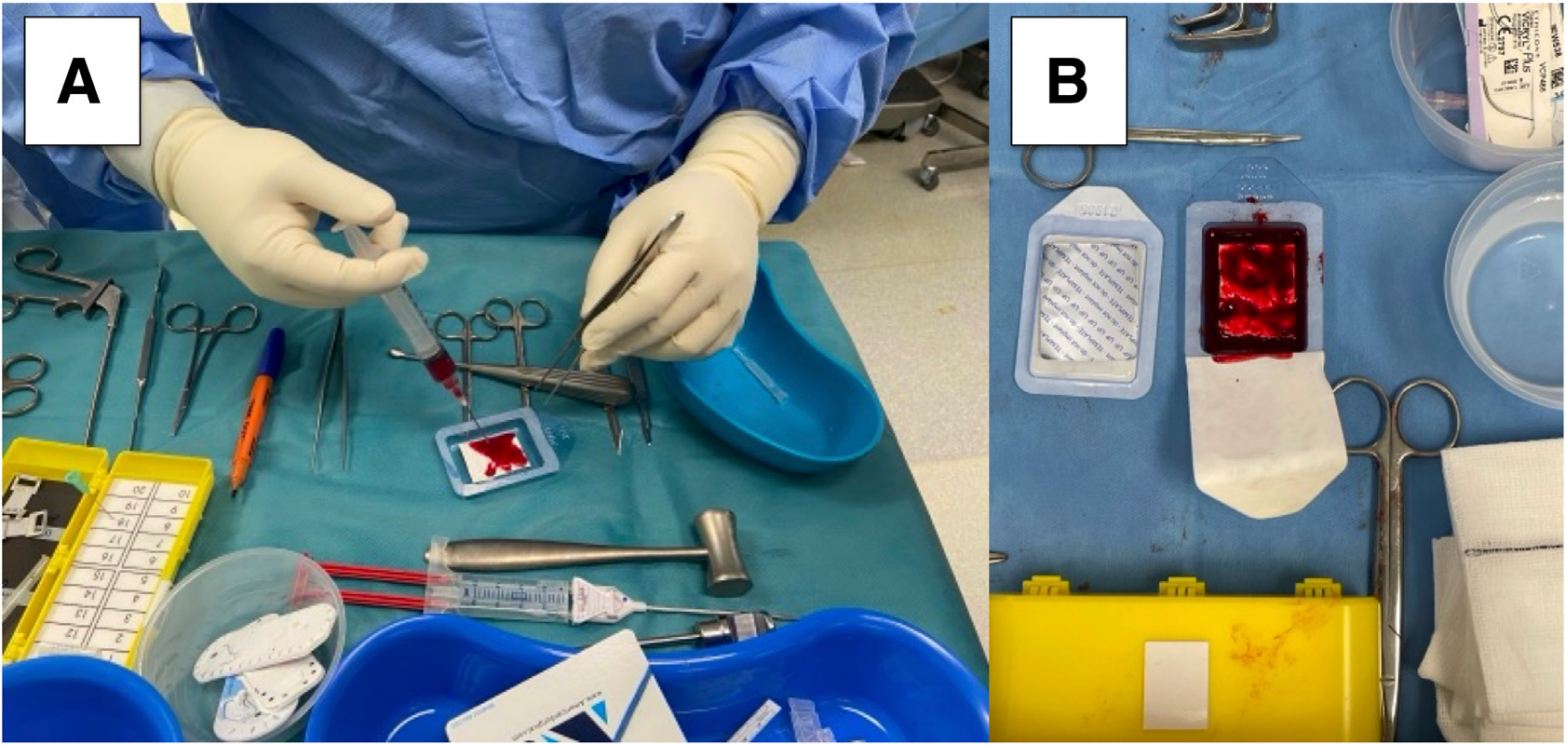
(A) Bone marrow aspirate concentrate is instilled onto the Chondro-Gide scaffold, which is left to soak. (B) The articular surface of the Chondro-Gide scaffold is marked for reference to allow for easy identification.

The articular surface of the Chondro-Gide is marked with a sterile marker for easy identification. The centrifuged BMAC solution is instilled into the Chondro-Gide within the sterile package container and left to soak for at least 5 minutes (Fig 2).

### Preparation of the Cartilage Lesion

**Fig 3:**
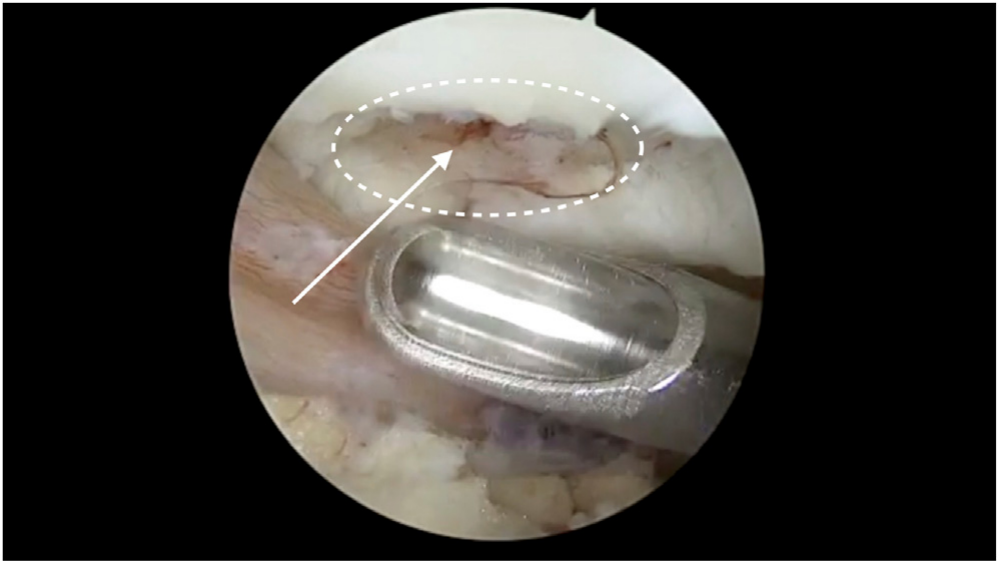
Characteristic punctate bleeding (arrow) on the defect bed of the right patella (circle) seen after adequate debridement until the articular tidemark.

We use the Chondrectom Extended set−big joints (Biovico, Gdynia, Poland) to debride the cartilage defect site. Sharp debridement is performed using the front and sideward chondrectomes, which allows the creation of a stable cartilage rim, a crucial step in successful cartilage repair. The parallel chondrectome is used to remove any intralesional osteophytes or calcified cartilage. A curette is used to debride the defect bed until the articular tidemark, using audiological and tactile cues as an indication of appropriate debridement back to healthy bone.^5^ Characteristic punctate bleeding indicates an adequate depth of debridement (Fig 3).

### Preparation of Minced Cartilage

**Fig 4:**
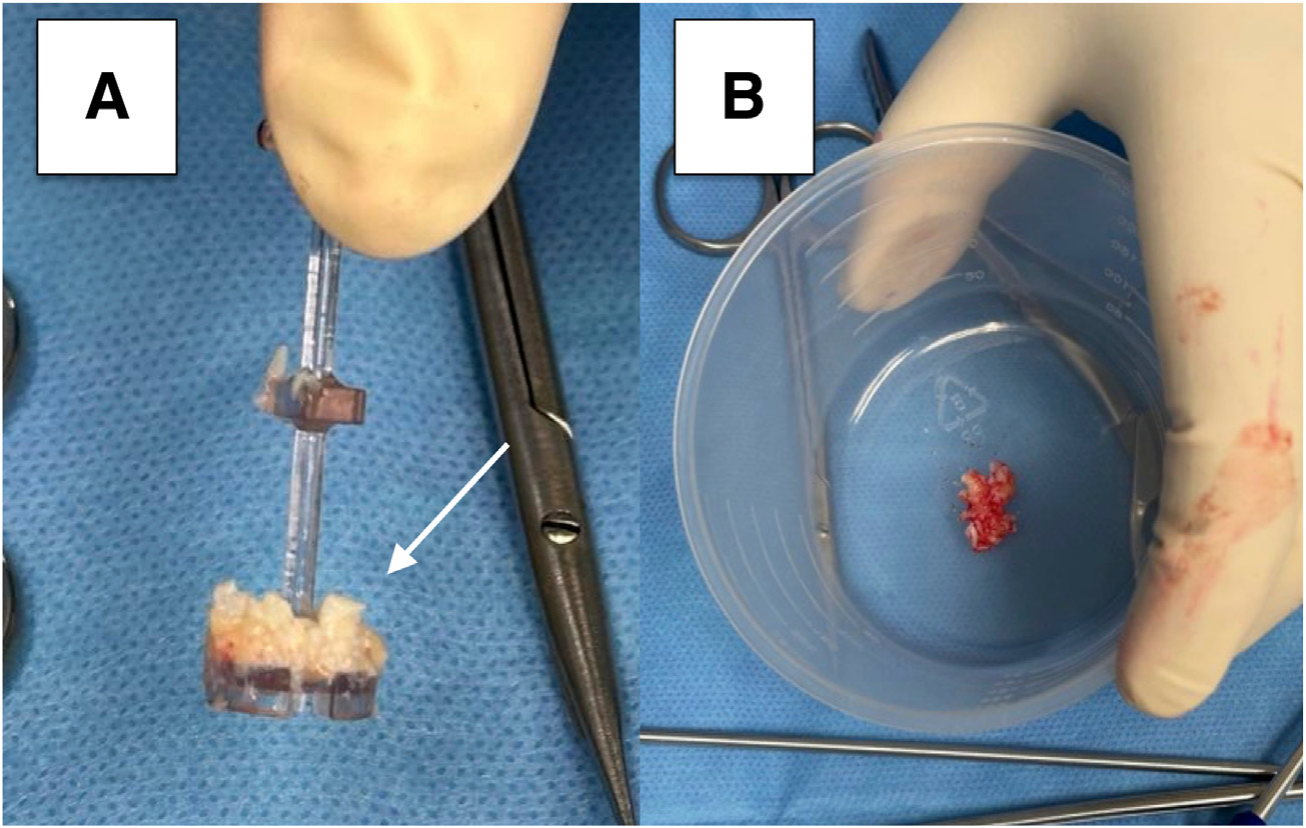
(A) Minced cartilage (arrow) collected in the collection basket via the GraftNet device. (B) The mixed cartilage is placed in a sterile receptacle and mixed with a few drops of the previously collected bone marrow aspirate concentrate.

A 4-mm bone cutter shaver blade on “forward” or “oscillate” mode is used to harvest autologous chondral fragments from non–load-bearing areas. The fragments are collected via the GraftNet (Arthrex, Naples, FL) autologous cartilage tissue collector, which is mounted between the shaver handpiece and suction tubing system. The collector has a filter chamber that collects the chondral fragments. This process avoids the manual mincing procedure previously used in open techniques.^6^ The minced cartilage is then placed in a sterile dish, and about 3 drops of BMAC are added in to form a cartilage paste (Fig 4). This paste is loaded into an application cannula and pushed to the tip using the trocar of the cannula. At this point, saline instillation is stopped, and dry arthroscopy conversion is achieved by suctioning of the remaining saline and introducing carbon dioxide gas into the knee.

### Graft Templating

The target lesion is dried with a gauze patty and the curved raspatory tool is used to approximate the size of the lesion. The BMAC-soaked is cut to the shape and size of the lesion using Metzenbaum scissors.

### Repair

**Fig 5:**
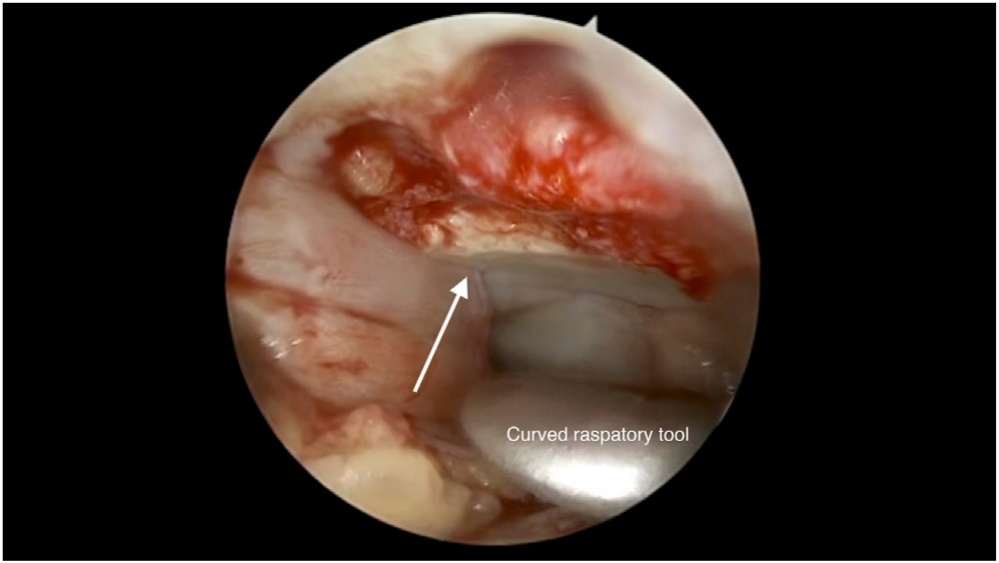
Minced cartilage and BMAC paste is delivered onto the defect site with a trocar. Pictured is the anterolateral portal arthroscopic view of the minced cartilage-BMAC paste in situ with Tisseel glue applied over the paste of the right patella (arrow). (BMAC, bone marrow aspirate concentrate.)

**Fig 6:**
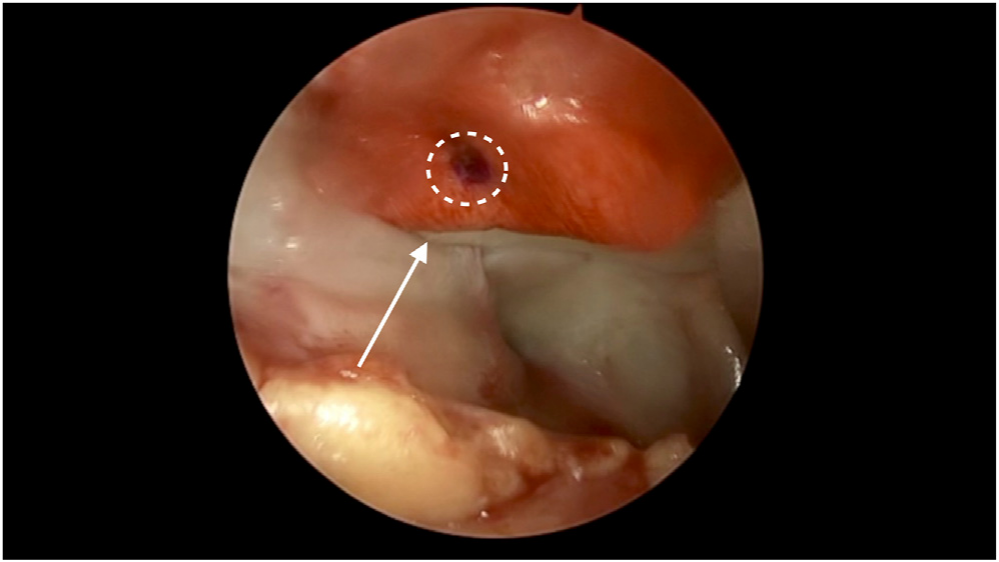
After a layer of Tisseel glue is applied over the minced cartilage–bone marrow aspirate concentrate paste in the right patella, the collagen membrane scaffold is introduced and placed over the glue and paste layers. Pictured is the graft in situ (arrow), with the markings (circle) made previously by the sterile skin marker on the scaffold seen.

**Fig 7:**
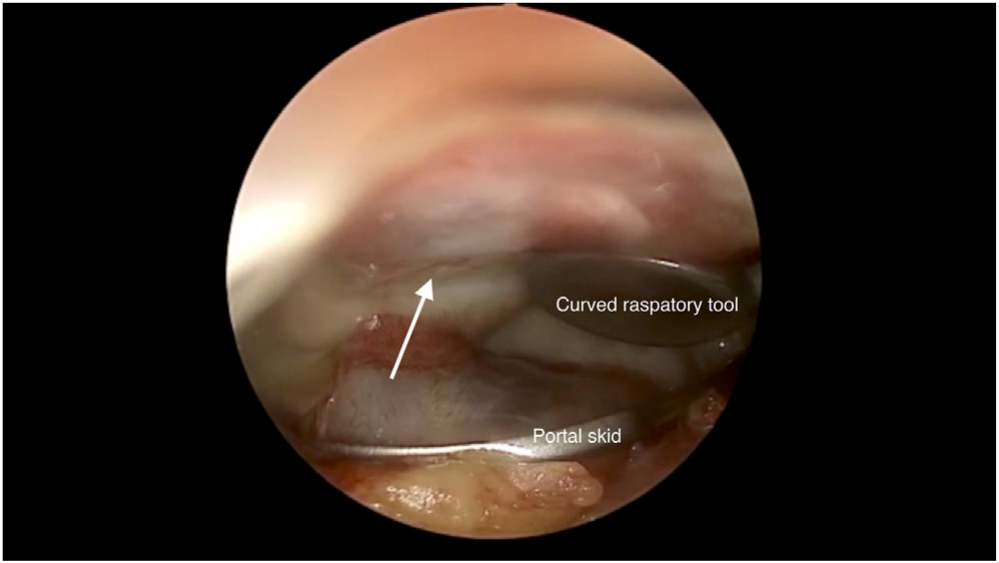
Pictured is the graft in situ with another layer of Tisseel glue applied (arrow) over the right patella repair. A curved raspatory tool is used to pat down and smoothen the graft over the lesion bed. A portal skid can also be used to push away any synovium that hinders visualization or interferes with graft placement.

The defect base is lined with a thin layer of fibrin sealant Tisseel (Baxter, Deerfield, IL), and left to harden for a minute. The minced cartilage mixed with BMAC is introduced above the hardened Tisseel layer with the application trocar. The minced cartilage layer is then sealed with another layer of Tisseel (Fig 5). Finally, the prepared Chondro-Gide piece that was cut to the dimensions of the defect is introduced with a pair of small artery forceps gently grasping the apex of the graft to prevent traumatizing the scaffold matrix (Fig 6). A layer of Tisseel glue is added to secure the scaffold in place. The curved raspatory tool or McDonald dissector is used to pat down and smoothen the graft onto the lesion bed (Fig 7). The final layer of Tisseel is then introduced and left to dry for at least 5 minutes.

After drying, the repair is palpated with the curved raspatory tool to assess for contact bleeding, which indicates inadequate drying. After confirmation of the absence of contact bleeding, the stability of the repair is evaluated. The knee is passively ranged through maximal flexion and extension through 10 cycles of motion. The arthroscope is then reintroduced into the joint to assess for any displacement or dislodgement. The procedure is converted back into wet arthroscopy with reintroduction of saline to further evaluate stability of the repair. Once the repair has been deemed satisfactory, arthroscopic fluid is then drained and all incisions are closed.

### Postoperative Protocol

The knee is fitted with an angle brace and locked-in extension. Continuous passive motion is crucial to minimize the risk of arthrofibrosis and is initiated on the first postoperative day.

## Discussion

The advantages/limitations and the pearls/pitfalls of the procedure are summarized in Table 1. We present specific techniques for patellar lesions to the previously described all-arthroscopic approach in a single-stage cartilage repair. The all-arthroscopic approach avoids the increased risks and disadvantages associated with arthrotomy, such as longer postoperative recovery time and more significant postoperative pain. The addition of minced cartilage enables support for the scaffold in lesions associated with significant subchondral bone loss to provide autologous reconstruction of the osteochondral unit.^7^ The literature has shown significant improvements in pain and function for open repair of patellar lesions with minced cartilage and Chondro-Gide at the 5-year mark,^3^ but more high-quality studies are required to study the long-term effects of this repair done arthroscopically. In conclusion, this single-stage, double-layered, cell-based cartilage repair procedure is a minimally invasive technique that can be used for cartilage lesions of the patella.

**Table 1:** Advantages, Limitations, Pearls, and Pitfalls

Advantages	• Single-staged surgery • Arthroscopic technique has lower morbidity and faster recovery compared with open procedures • Additional layers of cartilage repair provide additional stability and increased chance of successful repair
Limitations	• Increased intraoperative duration • Suboptimal visualization and technically difficult access via arthroscopy • Steep learning curve
Learning pearls	
For patellar lesions	• Place portals closer to the joint line for better lever arm
	• Create an accessory portal via a medial suprapatellar pouch approach to allow for easier access for debridement of the defect bed
	• Use one hand to manipulate and apply pressure to the patella while debriding the patellar lesion to allow for better control and tactile feedback; the arthroscope can be operated by the surgical assistant concurrently
For dry arthroscopy	• Insufflate joint with carbon dioxide at adequate pressure to prevent blood from pooling in the joint space
	• Use a metal suction tip or a portal skid as passive suction to the areas of fluid accumulation, where the pressure differential with push fluids through the suction tip
	• The portal skid can also be used to push away any synovium that hinders visualization or placement of the scaffold
For scaffold adhesion	• Keep the target defect bed as dry as possible with gauze patties before introducing the scaffold
	• The final repair should be sited within the defect cavity and not protrude above it
Pitfalls	• Scrape the walls of the cartilage fragment collector as the fragments tend to stick to the walls, giving the impression of inadequate collection when removing the collection basket • Mark the articular side of the scaffold to prevent confusion, as the scaffold is not symmetrical • Thoroughly wash the knee joint before transitioning to dry arthroscopy to dislodge any loose cartilage bodies from debridement

## Disclosures

All authors (E.J.X.Z., Z.G.W.O., E.V.L., I.D.D., and K.L.W.) declare that they have no known competing financial interests or personal relationships that could have appeared to influence the work reported in this paper.

## Supplementary Data

Video 1 Our technique of an all-arthroscopic single-staged cartilage repair procedure with minced cartilage and an acellular collagen I/III bilayer scaffold (Chondro-Gide) augmented with bone marrow aspirate concentrate in a patellar lesion.
